# P66 Multi-country synthesis of antimicrobial stewardship implementation in low- and middle-income countries: facilitators, barriers, and strategies across Bangladesh, Nigeria and India

**DOI:** 10.1093/jacamr/dlag102.072

**Published:** 2026-06-26

**Authors:** Shumonto Mowla Chowdhury, Omobolanle Margaret Abe, Rishal Dsouza, Rasha Abdelsalam Elshenawy

**Affiliations:** School of Health, Medicine and Life Sciences, University of Hertfordshire, UK; School of Health, Medicine and Life Sciences, University of Hertfordshire, UK; School of Health, Medicine and Life Sciences, University of Hertfordshire, UK; School of Health, Medicine and Life Sciences, University of Hertfordshire, UK

## Abstract

**Background:**

Antimicrobial Resistance (AMR) is a defining public health crisis of the twenty-first century, accounting for an estimated 1.27 million deaths annually, with low- and middle-income countries (LMICs) bearing the most severe consequences [1]. Whilst national action plans have multiplied considerably, evidence of durable policy implementation at the frontline remains limited. This study draws on three systematic reviews from Bangladesh, India, and Nigeria to critically examine the determinants of the sustained gap between stewardship policy and clinical practice [2].

**Methods:**

Three independent systematic reviews constituted the evidence base for this narrative synthesis, which was reported in accordance with PRISMA 2020 guidelines. Searches were conducted across MEDLINE (PubMed), Scopus, CINAHL, the Cochrane Library, Web of Science, and Google Scholar (2015–2025), using Boolean search strategies combining terms relating to antimicrobial stewardship, implementation, barriers, facilitators, and the respective country contexts of Bangladesh, India, and Nigeria. Quality assessment used the CASP checklist. Across three reviews, 38 studies were included, representing 39 042 participants across tertiary, secondary, primary, and dental care settings. Thematic analysis combined reported AMS implementation, prescribing practices, and facilitators and barriers, and cross-country comparative analysis revealed both universal and context-specific patterns.

**Results:**

Across 38 studies involving 39 042 participants, a clear and actionable evidence base was established to guide AMS strengthening across three LMIC contexts. Whilst core AMS strategies, such as multidisciplinary stewardship committees, prospective audit and feedback, and formulary restriction, remain inconsistently implemented, the findings reveal important and modifiable entry points for targeted, context-sensitive scale-up. In Bangladesh, high antibiotic exposure (over 70%) and low culture-guided prescribing (31%) highlight the transformative potential of diagnostic investment. In Nigeria, despite limited AMS awareness among 70% of healthcare professionals, the widespread presence of education programmes, prescribing guidelines, and committee structures in up to 80% of studies provides a strong foundation for sustainable stewardship. In India, growing recognition of inappropriate dental prescribing (over 50%) is driving demand for formal AMS frameworks. Across all three settings, consistently identified training deficits and policy-practice gaps define a clear reform agenda: strengthening diagnostics, institutionalizing accountability, and translating national action plans into frontline practice.

**Conclusions:**

Across the LMICs examined, this synthesis provides an evidence-informed foundation for meaningful AMS progress. Whilst implementation remains inconsistent, high clinician receptivity and existing policy frameworks create genuine opportunity for targeted reform. Education and training offer immediate entry points, but must be accompanied by investment in diagnostics, laboratory capacity, and institutional accountability. A phased pathway is recommended: beginning with surveillance and multidisciplinary team formation, progressing to audit and feedback mechanisms, and sustained by domestic financing. Critically, LMIC-specific stewardship models must be co-developed and validated with local stakeholders. These findings offer policymakers and healthcare leaders a clear roadmap to embed sustainable stewardship into routine clinical practice.
